# Birth Defects in Northern Iran (2008–2013)

**Published:** 2018-03

**Authors:** Arezoo MIRFAZELI, Nafiseh KAVIANY, Kanizreza HOSSEINPOOR, Mohammad ARYAIE, Mohammad Jafar GOLALIPOUR

**Affiliations:** Gorgan Congenital Malformations Research Center, Golestan University of Medical Sciences, Gorgan, Iran

**Keywords:** Birth defects, Gender, Ethnicity, Iran

## Abstract

**Background:**

Congenital anomalies are important medical and public health conditions. The pattern and prevalence of birth defects may vary over time or with geographical location. We investigated the live birth prevalence and occurrence pattern of birth defects in Golestan Province, northern Iran.

**Methods:**

This cross-sectional descriptive study was carried out on 144920 live newborns in 13 hospitals in Golestan Province, northern Iran, from 21 Jan 2008 to 19 Mar 2013. The newborns were examined for the presence of birth defects and mothers were interviewed for variables such as maternal age and ethnicity. In addition, data for each newborn was filed in a questionnaire and the coding of birth defects was translated to the International Classification of Diseases 10th revision–clinical modification (ICD-10-CM).

**Results:**

Overall, 1690 infants were diagnosed as having birth defects among 144920 live newborns. The prevalence rate of birth defects was 11.66 per 1000 live births, the prevalence of birth defects per 1000 was11.62 in males and 11.42 in females. The prevalence of congenital anomalies among native Fars, Turkmen and Sistani were 13.03, 11.16 and 13.07, respectively, per 1000 live births. Anomalies of the cardiovascular system were the most common defects; the prevalence rate of cardiovascular system was 8.34 per 1000 live birth.

**Conclusion:**

The prevalence rate of birth defects in this area was lower than in the other regions in Iran (20.3 per 1000 live births) but higher than in some parts of Asia (7.33 per 1000 live births).

## Introduction

With the promotion of immunization in the world, the mortality rate due to congenital malformations increased as the main cause of death (1). Moreover, birth defects can lead to prematurity, intrauterine growth retardation, morbidity, and mortality by increasing the age in future (1). Congenital malformations or birth defects are divided into minor and major according to structural, although can be presented as mental, behavior and metabolic disorders (2).

Factors involved in congenital anomalies include a plurality, gender, and parental factors such as ethnicity, socioeconomic status, and lifestyle, and drug use, medication during pregnancy, age, body weight, congenital diseases, and environmental exposure (2).

Birth defects take place in 20–30 per 1000 birth and multiple defects involved one-quarter of them. Severe birth defects are the one of the main cause of perinatal mortality, stillbirths, and abortions (3).

Birth defects are known to occur due to single gene defects, chromosomal abnormalities, environmental factors and multifactorial causes following reciprocal effects of environmental and genetic factors, but the causes of 60%–70% of birth defects have not been determined (3).

Birth defects vary according to geographical, economic, gender and ethnical conditions (2, 3). In 1986, the rates of joint dislocation, cleft lip, cleft palate and finger anomalies were investigated (4). The prevalence rates of birth defects have been reported from 10.1 to 17.7 per 1000 live births in previous studies (5, 6) in northern Iran and 7.33 to 30.57 per 1000 worldwide (2, 7).

In response to the new public health policy, the fortification of flour with folic acid, and the promotion of prenatal care in Iran, this study was undertaken to determine the proportion and pattern of congenital anomalies in live newborns in Golestan Province, northern Iran, during a 5-year period.

## Methods

This hospital-based cross-sectional study was carried out on 144920 live birth newborns in Golestan Province, northern Iran, during a 63-month period, from 1 Jan 2008 to 19 Mar 2013.

Ethical approval for the study was obtained from the ethics committee of Golestan University of Medical Sciences (Ethical code: 237192082611).

Golestan Province is one of the 31 provinces of Iran. Located in the north of the country, southeast of the Caspian Sea, it has a population of 1.6 million and an area of 20380 km^2^. Patients are usually from moderate to low socioeconomic class families with various ethnic backgrounds. Fars, Turkmen, and Sistani are the three main ethnic groups in the capital, Gorgan. Native Fars is the predominant group of inhabitants and has the most members, Turkmen is the ethnic group that emigrated from central Asia more than three centuries ago, and the Sistani group emigrated from southeastern Iran half a century ago.

All live newborns delivered in 13 hospitals throughout Golestan Province during the investigation were examined and screened for birth defects by pediatricians.

The parents of the newborns completed consent forms. Date of birth, gender and type of birth defects according to the International Classification of Diseases, (ICD-10) were recorded. Data for each newborn were filed in a questionnaire. The data were analyzed using SPSS software (ver.16 Chicago, IL, USA) and were compared with the Chi-square test. The 95% confidence interval for prevalence was estimated. A *P*-value of 0.05 or less was considered statistically significant.

## Results

The prevalence rate of birth defects was 11.66 per 1000 live births. The prevalence of birth defects was 11.42 per 1000 in females (RR=1.02 CI95%:0.93–1.12) and 11.62 per 1000 in males.

According to ethnicity, the prevalence of congenital anomalies among the native Fars, Turkmen and Sistani ethnic groups was 13.03, 11.16 and 13.07, respectively, per 1000 live births.

Some newborns had a multiplicity of malformations; therefore, the total number of congenital malformations overpassed the number of affected newborns. Totally, 2545 anomalies were documented in 1690 newborns with birth defects. Anomalies of the cardiovascular system were the most affected; the prevalence rate of cardiovascular system was 8.34 per 1000 live birth; among this group, the most frequent anomalies were ASD (3.06 per 1000 live birth). The musculoskeletal system anomalies were second in frequency; the prevalence rate of musculoskeletal system anomalies was 2.33 per 1000 live birth. Among this group, the most frequent anomalies were clubfoot (0.3 per 1000 live birth). Followed by, nervous system anomalies with the prevalence rate of 1.97 per 1000 live birth. In this group, the most frequent anomalies were congenital hydrocephalus (0.54 per 1000 live birth).

Besides, digestive system and Genitourinary System anomalies had the most frequent anomalies. The prevalence rate of anomalies in digestive system was 1.44 and Genitourinary System was 0.77 per 1000 live birth (Table 1).

**Table 1:** Frequency of Congenital Anomalies by System According to ICD-10 Classification in Northern Iran

***Malformation/System***	***Rate per 1000 Births***
The Cardiovascular System	8.34
The Musculoskeletal System	2.33
The Nervous System	1.97
Neural Tube Defects	1.02
The Digestive System	1.44
Genitourinary System	0.77
Chromosomal Abnormalities	0.66
Cleft lip and Cleft palate	0.35
Eye, Ear, Face, and Neck	0.32
The Respiratory System	0.18
Beta thalassemia	0.52
Hydrops fetalis	0.37
Other and unspecified anomalies	0.24

The relative risk of birth defects for the Turkmen to native Fars and Sistani to native Fars was determined to be 0.86 and 1.003, respectively. Based on the prevalence rate of birth defects in native Fars and Sistani ethnic groups were significantly higher than among Turkmen (Table 2).

**Table 2:** Association between gender, ethnicity, and birth defect prevalence

***Variable***	***Category***	***No. Newborns delivered***	***No. With birth defects***	***Rate per 1000 live births***	***Relative risk***	***95% CI***
Gender	Female	70349	804	11.42	-	-
	Male	74571	867	11.62	1.02	0.93–1.12
Ethnicity	Fars	60064	783	13.03	-	-
	Turkmen	48471	541	11.16	0.86	0.77–0.96
	Sistani	21867	286	13.07	1.003	0.88–1.15

## Discussion

In the present study, the prevalence of birth defects in live newborns was 11.66 per 1000 live births, which is comparable with our previous study from in northern Iran (6), which reported a prevalence of 17.7 per 1000 live births in 2007–2008. This reduction of the prevalence rate of birth defects can be due to the government health policy including folic acid consumption by young women, fortification of flour with folic acid, and the termination of pregnancy due to a major defect in the fetal period.

The prevalence rate of birth defects in our study was lower than Tabriz, north-west Iran, with 20.3 per 1000 live births (8) and Ahvaz, south-west Iran, with 20.2 per 1000 live births (9). Moreover, the total prevalence of congenital anomalies was 1.7 per 100 births, from 2000–2008 (10) and 165.5 per 10 000 births in 2007 (10). These rates of birth defects in Tabriz was higher than our result (11).

Different rates of birth defects in the world were present including Taiwan, 7.33 per 1000 births (2); two reports of China, 13.43 and 12.6 per 1000 births(12, 13); India, 22.2 per 1000 births (1); Egypt, 25 per 1000 births (14); Thailand, 26.12 per 1000 live births (15); Nigeria, 28 per 1000 births (16); Korea, 28.7 per 1000 live births (3) and Bradford, 30.57 per 1000 live births (17) (Table 3).

**Table 3:** Prevalence of birth defects in various areas in the world

***Author***	***Location***	***Time span of study***	***Prevalence per 1000 live births***
Chen (2)	Taiwan	2002	7.33
Present study	Gorgan, northern Iran	2008–2011	11.66
Yu (13)	China	2006–2012	12.6
Golalipour (6)	Gorgan, northern Iran	2007	17.7
Ahmadzadeh (9)	Ahvaz	2004–2006	20.2
Dastgiri (8)	Tabriz	2000–2011	20.3
Sarkar (1)	India	Sep 2011 to Aug 2012	22.2
El Koumi (14)	Egypt	2011	25
Pangkanon (15)	Thailand	2014	26.12
Obu (16)	Nigeria	2007–2011	28
Kim(3)	Korea	2005–2006	28.7
Sheridan (17)	Bradford	2007–2011	30.57

In our study, the prevalence rate of birth defects in male newborns was higher than that for females. This higher rate of birth defects in males has also been reported in other studies. Males were more affected than females (1.8:1) (7). Moreover, this rate was similar to other reports from Iran (9).

According to ethnicity, the rate of birth defects in this study was 13.03, 11.16 and 13.07 per 1000 live births for native Fars, Turkmen, and Sistani, respectively. In this regard, other studies have reported that ethnicity has an impact on the prevalence of birth defects (12, 17).

The risk of birth defects was greater for mothers of Pakistani origin than for those of white British origin (univariate RR 1·96, 95% CI 1.56–2.46) (17). With regard to the pattern of birth defects in this study, the most common system involved was the cardiovascular system, followed by the musculoskeletal system, nervous system, digestive system, genitourinary system and chromosomal defects, which is in contrast with the study reported from Egypt (7). In Egyptians, the common congenital malformations in newborns were an abnormality in CNS, genitourinary system and genetic disorders (7).

However, some studies have reported (1, 3, 9) musculoskeletal system defects as the highest. In a study in Taiwan (2), nervous system, eye, and face, cardiovascular system, digestive system, urogenital and musculoskeletal system anomalies were common. The genitourinary tract and kidney defects, anomalies of the nervous system, and limb anomalies accounted proportionally for more than 68% of anomalies in the Tabriz (10).

In a study in Birjand in Southeast of Iran, the rate of nervous system anomalies was 11.23% (18). In addition, the rate of NTDs was 5.01 per 1000 in Farhud study in Hamadan from 1991 to 1997 (19). Although this was a hospital-based study, the majority of deliveries in Golestan Province take place in the 13 hospitals, so the data can be taken as a good reflection of the birth defects in the area. However, we were not able to use the data from three hospitals in Golestan Province.

## Conclusion

The prevalence rate of birth defects in this area was lower than in the other regions in Iran (20.3 per 1000 live births) but higher than in some areas of Asia (7.33 per 1000 live births). Inadequate diagnosis of birth defects at birth and health policy can be considered the possible reasons for the difference in the prevalence of birth defects between our region and other parts of the world, although, in recent years, this diagnosis has been facilitated more than in past years.

## Ethical considerations

Ethical issues (Including plagiarism, informed consent, misconduct, data fabrication and/or falsification, double publication and/or submission, redundancy, etc.) have been completely observed by the authors.
